# Sexual dysfunctions among men with paroxysmal or persistent atrial fibrillation - a two-center study in the Polish population

**DOI:** 10.1192/j.eurpsy.2022.724

**Published:** 2022-09-01

**Authors:** A. Szczegielniak, J. Smolarczyk- Kosowska, M. Wybraniec, M. Krzystanek

**Affiliations:** 1 Medical University of Silesia, Department Of Psychoprophylaxis, Katowice, Poland; 2 Medical University of Silesia, First Department Of Cardiology, Katowice, Poland; 3 Medical University of Silesia, Department And Clinic Of Psychiatric Rehabilitation, Katowice, Poland

**Keywords:** sexual dysfunction, atrial fibrillation, CSFQ-14

## Abstract

**Introduction:**

There are no comprehensive studies on sexual dysfunctions among people with paroxysmal and persistent atrial fibrillation after excluding concomitant somatic and mental disorders, even though their presence has a significant impact on observed changes in sexual activity and behaviour. Most of the available studies among males are focused on erectile dysfunction due to well established relationship of it with the occurrence of cardiovascular disorders in the future.

**Objectives:**

The aim of the study was to assess the prevalence of sexual disorders among male patients treated for paroxysmal and persistent atrial fibrillation.

**Methods:**

The study group included 54 men diagnosed with paroxysmal and persistent atrial fibrillation, qualified for electrical cardioversion and/or ablation of circumferential pulmonary vein, not burdened with additional somatic diseases. The control group consisted of 55 men matched in terms of sex, age and health condition. The study used standard CSFQ-14 sexuality assessment questionnaires, and the WHOQoL-BREF quality of life survey. Mental status examination was performed to exclude those with mental disorders, currently or in the past receiving psychiatric and/or addiction treatment.

**Results:**

The analysis of the survey studies showed that among the surveyed men with paroxysmal and persistent atrial fibrillation, the prevalence of sexual problems is high (61.1% study group vs 47.3% control group). There were significant differences between the study group with lower sexual function scores in pleasure, desire/interest, arousal/erection, orgasm/ejaculation, and in the overall CSFQ-14’s score. The desire/frequency ratio did not differ between the groups.

**Conclusions:**

Comprehensive care requires the assessment of sexual satisfaction and the presence of possible dysfunctions using standardized tools.

**Disclosure:**

No significant relationships.

